# Penalty shoot-outs are tough, but the alternating order is fair

**DOI:** 10.1371/journal.pone.0315017

**Published:** 2024-12-09

**Authors:** Silvan Vollmer, David Schoch, Ulrik Brandes

**Affiliations:** 1 Department of Mathematical Sciences, University of Copenhagen, Copenhagen, Denmark; 2 Department of Computational Social Science, GESIS, Cologne, Germany; 3 Department of Humanities, Social and Political Sciences, ETH Zurich, Zurich, Switzerland; Mugla Sitki Kocman University: Mugla Sitki Kocman Universitesi, TÜRKIYE

## Abstract

We compare conversion rates of association football (soccer) penalties during regulation or extra time with those during shoot-outs. Our data consists of roughly 50,000 penalties from the eleven most recent seasons in European men’s football competitions. About one third of the penalties are from more than 1,500 penalty shoot-outs. We find that shoot-out conversion rates are significantly lower, even for regular penalty takers, and attribute this to worse performance of shooters rather than better performance of goalkeepers. We also find that, statistically, there is no advantage for either team in the usual alternating shooting order. Complemented by a number of detailed observations, these main findings underline that penalty shoot-outs represent a different condition requiring dedicated means of training and coaching.

## Introduction

Association football (soccer) is a low-scoring game, with typically less than three goals per match [1]. On average, one in three matches sees a penalty awarded, and more than three in four of these lead to a goal. Moreover, since their adoption by the International Football Association Board (IFAB) in 1970, penalty shoot-outs are the ultimate method of choice to break ties in competitions with elimination rounds. Penalties are therefore an important element of the game, both during regulation and in shoot-outs.

Research into factors influencing the outcome of a penalty abounds. It includes findings on player nationality [2], player status [3], penalty importance [4], the target zone of a shot [5–7], run-up length, strike type, footedness, match status [6] and other circumstantial factors [6–8]. Specifically for penalty shoot-outs, the impact of a team’s league [9], team strength and match venue [10] have been considered.

Our focus, however, is not on strategies and factors influencing success, but on empirical regularities of penalty conversion rates in the in-game and shoot-out conditions. In other words, we do not intend to identify success factors, but to assess whether there are observable outcome differences associated with the two major settings in which penalties are taken.

A majority of prior studies indeed observe higher conversion rates for in-game penalties [2, 11, 12] compared to shoot-out penalties [13]. Based on a much larger and more recent dataset, we not only confirm this finding, but reveal that this is almost entirely due to a larger number of misses.

A hotly debated issue for shoot-outs is whether the common alternating order in which penalties are taken favors the team going first. A number of models suggest that, in theory, the current order is unfair, because a team lagging behind faces pressure to draw level so that the beginning team is likely to enjoy a first-mover advantage [14–18]. Empirically, however, the evidence is more controversial. While several studies do find a bias [19–22] others do not [8, 23–25]. The most recent of these studies is based on all shoot-outs in all major tournaments of the past fifty years, and sides with the latter group [25]. From even more and crucially also more recent data we conclude that, at least in men’s football, no discernible bias results from the alternating order. Indeed, we only consider the men’s game, because it has been suggested that the mechanisms governing shoot-out results in the women’s game differ substantially [25].

## Materials and methods

Our data consists of penalties from 115 major national and international football competitions throughout Europe. It has been scraped from Transfermarkt, a commercial website with comprehensive and up-to-date information on players, clubs, and competitions.

The data was retrieved in July 2023 for the past eleven seasons (2012–13 to 2022–23, starting with EURO 2012) of men’s competitions run by either UEFA or one of its 55 member associations. A total of 54 first-division leagues constitute the highest levels of professional football in each of the associations (Liechtenstein has no league of its own), and a total of 54 national cup competitions add some breadth (Transfermarkt does not cover cup competitions for North Macedonia and for Montenegro, but for England we include two, the FA Cup and League Cup). In addition to six international competitions organized by UEFA (European Championship, Nations League, Champions League, Europa League, Europa Conference League, and Super Cup), we include the FIFA World Cup Finals of 2014, 2018, and 2022, where many of the non-European teams field players playing for European clubs. With almost every league accompanied by a cup, more than half of the competitions feature elimination rounds in which ties are ultimately resolved by penalty shoot-outs.

**Fig 1:**
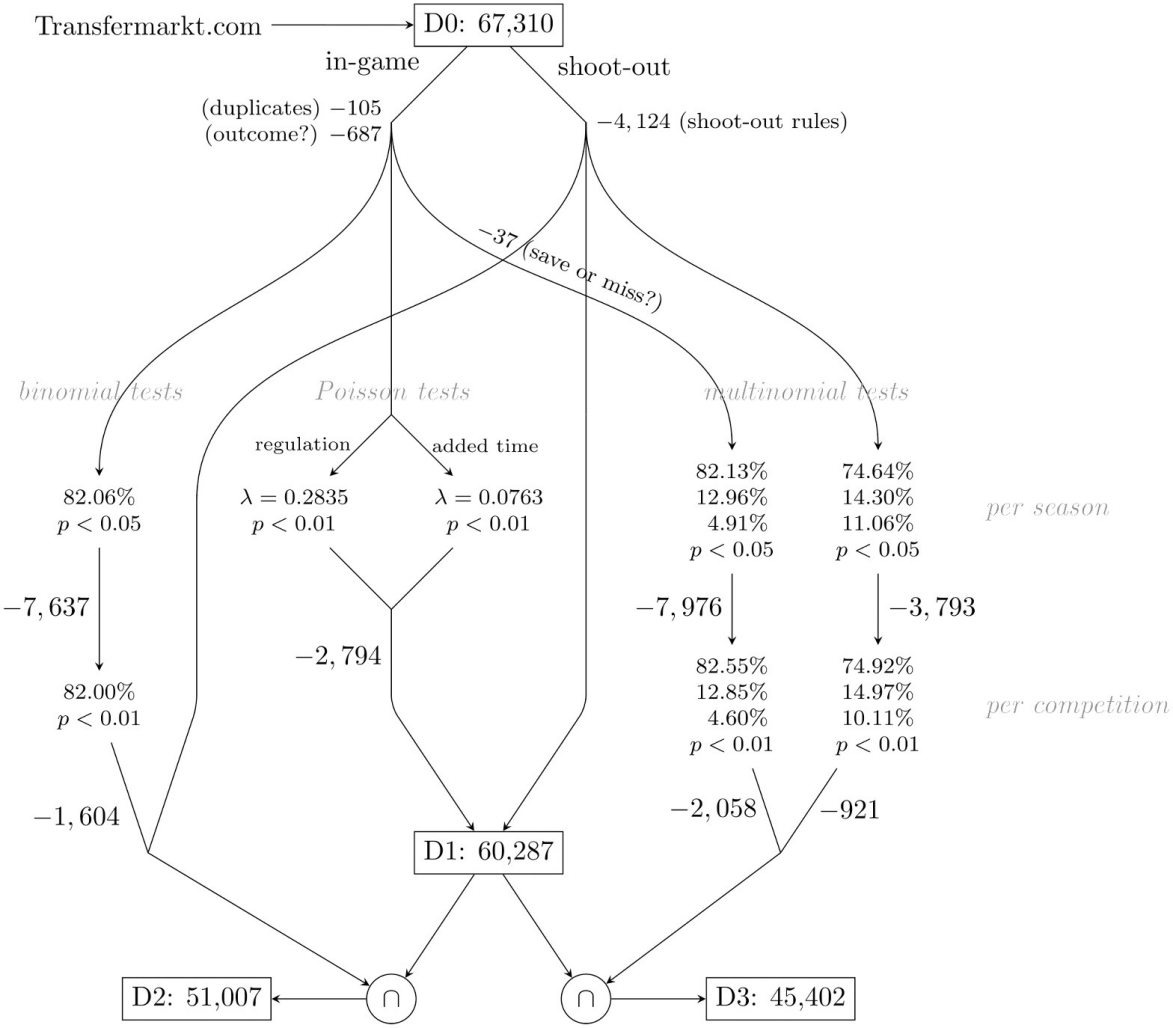
Preparation of datasets. Assuming systematic reporting issues, seasons of a competition, or entire competitions (eleven seasons) are removed, if frequency tests flag them as outliers. Further details are given in the main text.

The parsing of match summaries from 1,126 pairs of competitions and seasons yielded a total of 67,310 reported penalties. We refer to this raw data as dataset D0. Match data published by Transfermarkt is not necessarily sourced from official records, and we cannot rely on its accuracy, completeness, or consistency. We attempt to mitigate possible effects of apparent or suspected reporting issues by removing data of dubious quality as sketched in Fig 1. The majority of these cleaning steps rely on outlier detection using Poisson, binomial, and multinomial significance tests and are described in more detail below.

### Penalty occurrence rates (D1)

In a first step, the raw dataset D0 is filtered for apparent inconsistencies and suspected reporting biases. Not only are non-shoot-out penalties subject to a wider variety of reporting issues, the rules for penalty shoot-outs also make it easier to spot inconsistencies. The following two sets of filters have been applied to remove potentially problematic cases.

**In-game (regulation and extra time) penalties**. By querying in-game penalties for the same team within minutes, we identified 105 in-game penalties that were deemed double entries, generally because a goal was scored from a rebound. After manually consolidating these, we turned our attention to potential under-reporting. Especially in niche competitions, penalties are sometimes included only if they led to a goal, or goals are recorded without indication that they resulted from a penalty. We therefore applied frequency tests to eliminate seasons of competitions that featured significantly fewer penalties than the others. Based on the respective numbers of matches and penalties, we estimated occurrence rates of 0.2835 penalties during regulation and 0.0763 during extra time. A one-sided Poisson test at the 1% level flagged 108 seasons across 45 different competitions in which in-game penalties may have been under-reported. We therefore dropped these seasons from the dataset, resulting in 2,794 fewer in-game penalties.
**Shoot-out penalties**. Shoot-outs follow procedural rules which are helpful in identifying inconsistencies. According to the IFAB Laws of the Game [26] five penalties are taken by each team in alternating order. A shoot-out is terminated early if a team can no longer level the score with the remaining penalties, and it is continued beyond the first ten attempts with one additional penalty for each team as long as the score remains tied. An entire shoot-out is therefore removed from the dataset, if the order between the teams is not alternating (some competitions have indeed experimented with other formats), the number of penalties is less than six or an odd number larger than ten, all of its penalties resulted in a goal, or the final score is a draw.
  A total of 467 shoot-outs in D0 have inconsistent data, leading to the removal of 4,124 penalties.

Having removed shoot-outs and seasons of competitions with clearly or potentially inconsistent reporting, we are left with an initial dataset, D1, of 60,287 penalties. Further quality checks are applied to build the two main dataset used in our study. They differ by the level of detail required for the outcome of a penalty.

### Two-way outcomes: Goal or no goal (D2)

For analyses that require only knowledge about whether a penalty kick resulted in a goal or not, we still need to ensure that unsuccessful kicks are indeed reported. In parallel to the overall rate by which penalties are awarded, we therefore applied an additional frequency test for in-game penalty outcomes.

Starting from the full scraped dataset D0, 687 penalties with missing or indeterminate outcomes are excluded. From the remaining in-game penalties, we obtained an initial estimate of 82.06% for the in-game penalty conversion rate and applied a binomial test to filter out time periods with seemingly lower reporting standards. A total of 7,637 penalties were taken in seasons with significantly different conversion rates (5% level). After excluding these, the initial estimate for the in-game conversion rate is revised to 82.00%. A second, stricter, binomial test is then applied to flag systematic reporting issues for entire competitions in which the accumulated conversion rates over all eleven seasons deviate significantly on the 1% level. Dropping these competitions excludes another 1,604 penalties. The two tests combined thus led to the exclusion of 9,241 penalties from 231 seasons across 64 different competitions.

Taking into account that some of the flagged penalties have already been marked by the Poisson test for in-game penalty occurrence (rather than conversion) rates, the resulting dataset D2 to be used in analyses involving binary outcomes contains a total of 51,007 penalties.

### Three-way outcomes: Goals, saves, and misses (D3)

In some analyses, we are also interested in the different rates by which penalties are saved by the goalkeeper or missed by the kicker. The differentiation between these two non-goal outcomes presents additional sources of error in the scraped data.

Starting from the scraped data D0, we again eliminated the 687 in-game penalties with incomplete outcomes and an additional 37 non-goal penalties without distinction between the two possible reasons. The outcome labels for the remaining 62,357 penalties are normalized to either goal, saved, or missed with estimated rates of 82.13% (goal), 12.96% (saved), and 4.91% (missed) for in-game penalties, and 74.64% (goal), 14.30% (saved), and 11.06% (missed) for shoot-outs. Multinomial tests are then applied to identify further reporting biases such as non-goal penalties invariably reported as missed.

The season-wise 5%-level significance test flagged a combined number of 7,976 in-game penalties and 3,793 shoot-out penalties. Revised outcome-rate estimates after their exclusion are 82.55% (goal), 12.85% (saved), and 4.60% (missed) for in-game penalties, and 74.92% (goal), 14.97% (saved), and 10.11% (missed) for shoot-outs. The subsequent 1%-level significance test for competitions over the entire eleven seasons flagged a combined number of 2,058 in-game penalties and 921 shoot-out penalties.

In total, 14,748 penalties from 279 seasons across 82 different competitions were flagged. Excluding these penalties from the initial dataset, again in combination with the Poisson test for penalty occurrence rates, yields a dataset D3 comprised of 45,402 penalties with plausibly recorded three-way outcomes.

### Summary and data analysis

From the above cleaning and plausibility filtering steps we obtain two main datasets for this study, D2 and D3. Case numbers and available attributes are summarized in Tables 1 and 2.

**Table 1:** Description of penalty datasets.

datasets	penalties	regulation	extra	in-game	shoot-out
D0: as scraped	67,310	43,845	446	44,291	23,019
D1: penalty occurrences	60,287	40,960	424	41,384	18,903
D2: two-way outcomes	51,007	31,780	324	32,104	18,903
D3: three-way outcomes	45,402	30,908	305	31,213	14,189

Data scraped from Transfermarkt for the 2012/13–2022/23 seasons has been filtered to exclude seasons and competitions with implausible occurrence rates and outcome distributions attributed to inconsistent reporting.

**Table 2:** Attributes in datasets D2, D3 (seasons 2012–13 to 2022–23).

attributes	values
competition	first divisions in 54 UEFA member countries, 54 cup national competitions UEFA Champions/Europa/Conference League, Super Cup UEFA EUROs 2012, 2016, 2020; Nations League FIFA World Cup Finals 2014, 2018, 2022
season	from 2012/2013 to 2022/2023
match	participating teams
result	final match outcome
setting	in-game (regulation or extra time) vs. shoot-out
time	minute for in-game, position in shooting order for shoot-out
players	penalty taker and goalkeeper (with their respective clubs/countries)
score	at which the penalty was taken
outcome	goal/no goal in D2 and goal/save/miss in D3

The bulk of our analysis consists of descriptive statistics on penalty outcomes, segmented according to

- penalty condition (in-game and shoot-out),
- players involved (kickers, goalkeepers, and teams), and
- moments in time (in-game minutes and shoot-out order)

Binary logistic regression is employed to determine whether factors other than penalty condition influence success rates, and the cumulative Poisson binomial distribution is used to establish a baseline for shoot-out winning probabilities.

All data collection, preparation, and analysis procedures have been scripted using standard functions available in the R programming environment [27]. Charts in this article have been purpose-build using the Ti*k*Z graphics package for LATEX [28].

## Results

In both D2 and D3, roughly one third of the penalties are from shoot-outs. Home teams are awarded 55.82% (55.96%) of all in-game penalties in dataset D2 (D3), for a strikingly high advantage of more than eleven percentage points. Across all 115 competitions and eleven seasons we identified 21,948 (18,815) unique penalty takers and 7,196 (6,810) unique goalkeepers in the two-way (three-way) dataset. For 1,112 (876) penalties in D2 (D3), information on the goalkeeper is missing.

### Outcomes by penalty condition

**Fig 2:**
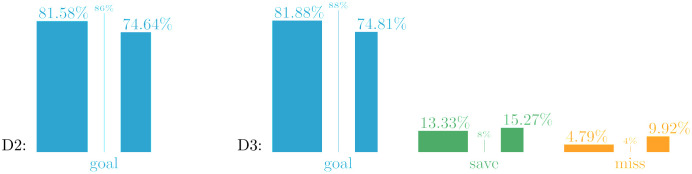
Relative frequencies in two-way and three-way datasets D2 and D3. Outcomes are split by match period (regulation, extra time, shoot-out). Widths of bars are proportional to percentage of penalties in the respective period, showing that numbers during extra time are too small to be relevant. We therefore combine regulation and extra time into in-game penalties.

Fig 2 summarizes conversion rates by match period. These are broken down further by competition format in Tables 3 (cups) and 4 (leagues). The common label “Big-5” refers to the first tiers in England, Spain, Italy, Germany, and France, which are generally considered to be the top leagues within UEFA, a sentiment corroborated by the averages of the UEFA Country Coefficients during the seasons from 2012–13 to 2022–23.

**Table 3:** Outcome rates for cup competitions in D3.

		in-game (regulation and extra time)				shoot-out			
	cups	penalties	goal	save	miss	penalties	goal	save	miss
national	46	6,615	84.93%	11.07%	4.01%	13,739	74.79%	15.21%	9.99%
international (clubs)	4	863	80.88%	13.21%	5.91%	256	79.30%	15.23%	5.47%
international (selections)	3	216	80.09%	13.43%	6.48%	194	70.10%	19.59%	10.31%
combined	53	7,694	84.34%	11.37%	4.29%	14,189	74.81%	15.27%	9.92%

Rates fluctuate where numbers are small, but overall in-game goal probabilities are even higher than in league competitions.

**Table 4:** Outcome rates for leagues in D2 and D3.

	D2			D3				
	leagues	penalties	goal	leagues	penalties	goal	save	miss
Big-5	5	4,500	81.04%	5	4,034	81.26%	13.78%	4.96%
other leagues	48	19,820	80.95%	47	19,485	81.14%	13.92%	4.93%
combined	53	24,320	80.97%	52	23,519	81.16%	13.90%	4.94%

Rates do not differ between the Big-5 and other leagues. All penalties have been taken during regulation as there are neither extra time nor shoot-outs in these competitions.

As international cup competitions often feature a group stage, we tested whether in-game penalty conversion rates differ between group and elimination rounds, where stakes may be more comparable to shoot-out situations. After excluding penalties during group stages from Table 3, 273 and 32 in-game penalties remain for clubs and national teams. While these are too few to draw reliable conclusions, conversion rates of 82.05% and 90.62% show no difference to other in-game situations, further emphasizing the exceptionality of the shoot-out condition.

**Fig 3:**
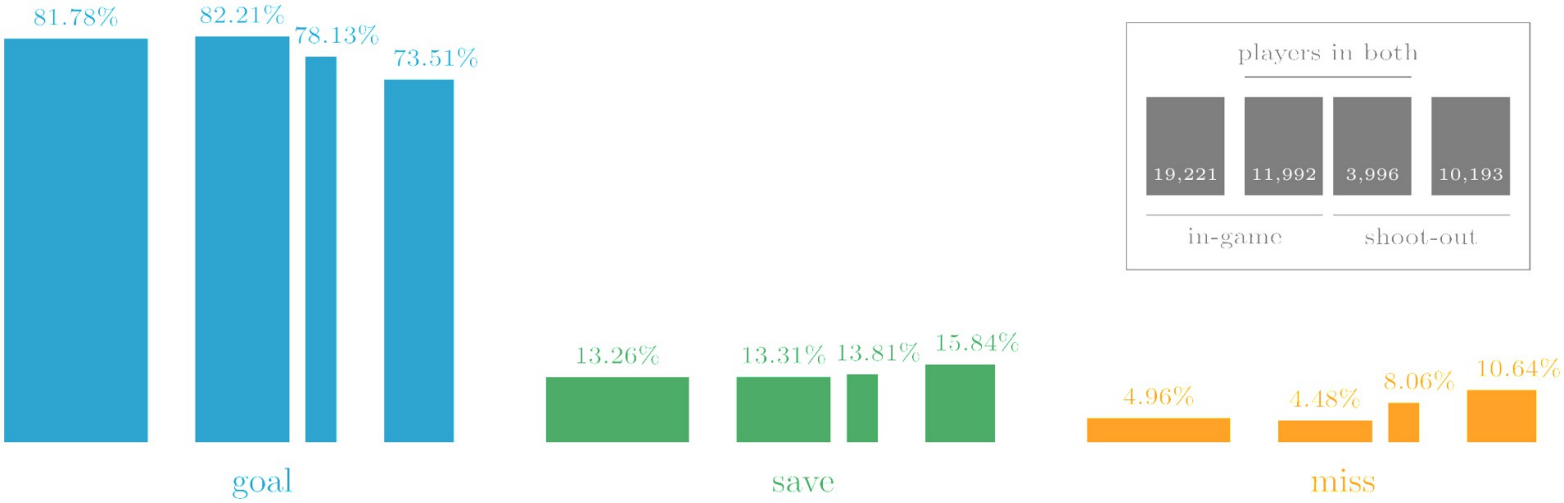
Relative frequencies in D3. Outcomes are split by condition players appear in. Only 2,855 players have taken both in-game and shoot-out penalties. Bar widths are proportional to the number of penalties.

A rule-induced difference between penalties during regulation and in shoot-outs is that penalty kickers can be chosen freely during a match, whereas in shoot-outs players can be nominated for another penalty only after all eligible player have take a turn. Fig 3 summarizes the performance of penalty takers split into three groups: players taking only in-game penalties, players taking only shoot-out penalties, and those taking both. For the last group, performance is given separately for both conditions.

Binary logistic regression is applied to identify possibly confounding factors. The outcome variable is goal or no goal, and the binary fixed effects listed in Table 5 are included. The reference level (all binary variables equal to zero) is an in-game penalty in a national league, taken by a player of the away team with the experience of one in-game and one shoot-out penalty. A kicker is considered to appear at the mark repeatedly, if he has taken penalties in both conditions with more than one penalty in at least one of them. We included seasons by the year they start in and ignored that in a comparatively small number of cases such as international tournaments and international cup finals in which a team labeled home team is not actually playing at their home ground.

**Table 5:** Binary logistic regression of penalty outcomes in D2.

variable	estimate	exp^estimate^	std. error	z value	p^†^
(intercept)	-10.477	0.0000	7.4752	-1.4016	0.6442
shoot-out ***	-0.3867	0.6793	0.0498	-7.7608	0.0000
players					
repeated appearance ***	0.3056	1.3574	0.0583	5.2378	0.0000
only in-game ***	0.2232	1.2501	0.0610	3.6578	0.0018
only shoot-out	-0.0321	0.9684	0.0600	-0.5351	0.9769
competition					
national cup ***	0.2352	1.2651	0.0364	6.4552	0.0000
international cup	0.0543	1.0558	0.0784	0.6928	0.9769
international selections	-0.1311	0.8771	0.1127	-1.1634	0.7340
environment					
home team	-0.0381	0.9626	0.0220	-1.7335	0.4981
season	0.0058	1.0058	0.0037	1.5645	0.5885

*p < 0.1

**p < 0.05

***p < 0.01

^†^ Bonferroni-Holm adjusted

All variables are coded as binary variables except for the season.

While home teams are awarded more penalties, there is no significant difference in conversion rates between home and away team. We explicitly compare rates in Table 6. Rejection of the idea that crowds tend to influence outcomes is corroborated by isolating a one-year period largely played without fan attendance from the end of the 2019/2020 season to the end of the 2020/2021 season because of COVID-19 regulations. Home and away outcome rates are essentially unaffected during this time.

**Table 6:** Outcomes for in-game penalties in leagues in D3.

	home teams				away teams			
	penalties	goal	save	miss	penalties	goal	save	miss
20/21 only	3,254	81.50%	13.40%	5.10%	2,675	81.72%	13.79%	4.49%
without 20/21	10,265	80.83%	14.32%	4.85%	7,325	81.28%	13.57%	5.15%

Outcomes are split by home and away team and by a one year period generally played without stadium crowds.

### Outcomes by players involved

The analysis is broken down further by investigating the performance of individuals and teams most frequently involved in penalties. To compile the following lists of penalty takers, goalkeepers, and teams filtering thresholds for the number of penalties have been set such that at least 50 players or teams are selected into a list. In case of a tie for the 50th rank, all players or teams with the same number of appearances are included.

#### Penalty takers

**Fig 4:**
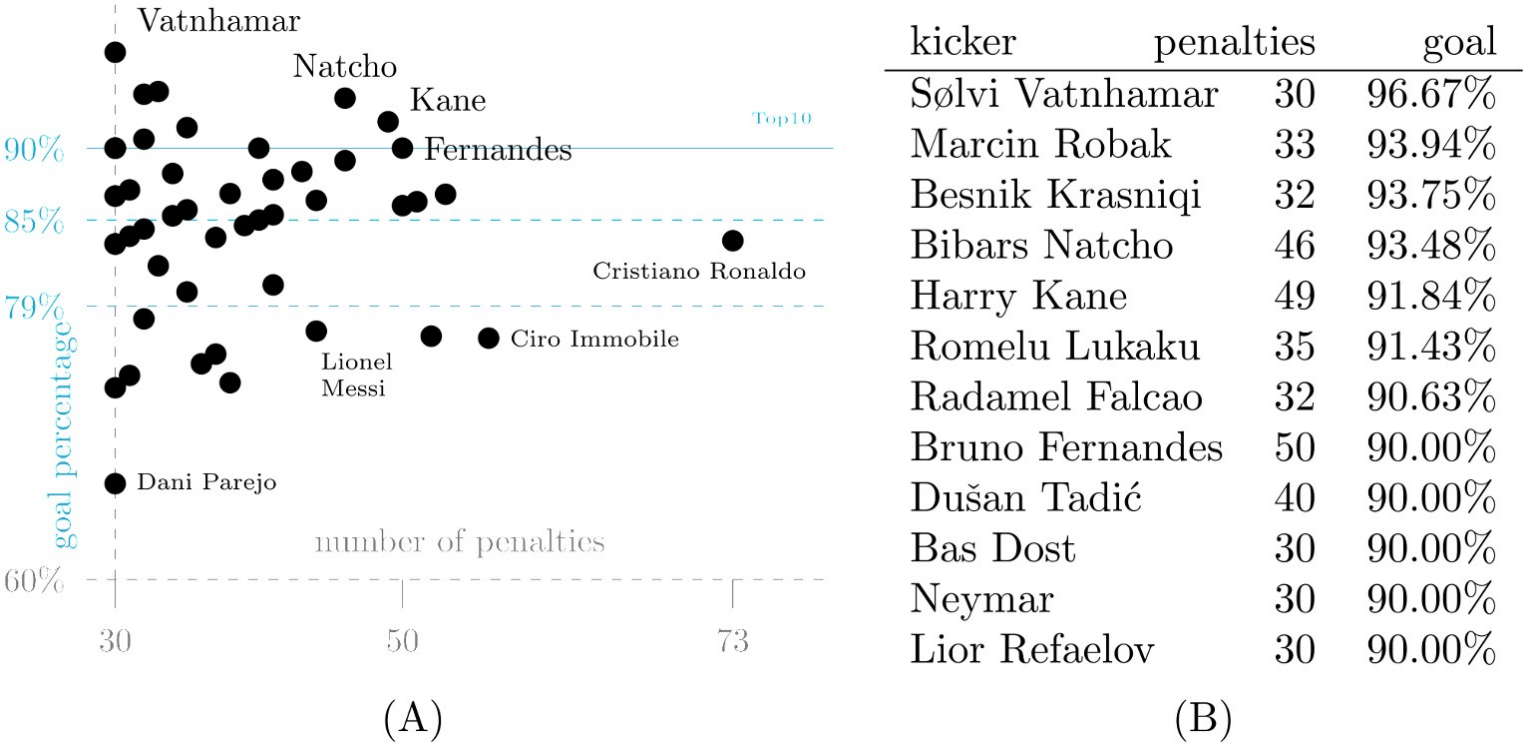
Most frequent penalty kickers in dataset D2. There are 51 players with 30 or more attempts. Across all competitions and match periods, 79% of penalties in D2 result in a goal. A: Goal percentages of frequent kickers. B: Top10+2 in goal percentage.

It would seem rational that the best kickers appear most often at the mark, because better teams win more penalties and have better kickers to execute them. A threshold of at least 30 penalties yields 51 penalty takers appearing most frequently in dataset D2, and success rates are given in Fig 4. Their collective conversion rate of 84.77% compares favorably to the 78.81% of all others (for an overall total of 79.04%), suggesting that teams do tend to select from their most dependable kickers.

Over the past eleven seasons, Sølvi Vatnhamar is the most successful high-volume penalty taker, converting all but one of his 30 penalties for Víkingur Gøta in the Faroe Islands Premier League. A different player who converted all but one of his attempts, but does not appear in the above list, is Max Kruse. For five different clubs he has taken a total of 27 penalties and therefore did not make the threshold.

The player who took by far the most penalties, Cristiano Ronaldo, has a success rate just below the average of all frequent kickers, whereas another frequent penalty taker, Lionel Messi, succeeded just below the overall average. Given his low goal percentage of 67%, it is surprising that Dani Parejo is among the most frequent penalty takers.

We do not distinguish between in-game and shoot-out penalties, because players have too few shoot-out appearances for analysis. The maximum is attained by Jorginho, who has taken eleven shoot-out penalties (and converted nine), and for the next three players (James Maddison, Mason Mount, and Paulinho) this number is already down to eight.

#### Goalkeepers

**Fig 5:**
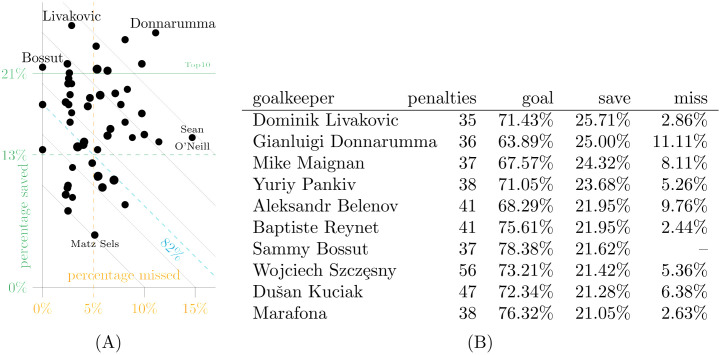
Goalkeepers most frequently facing in-game penalties in dataset D3. There are 51 goalkeepers who faced 34 or more in-game penalties, and dot size in the scatterplot on the left varies with that number. The goal rate (percentage neither saved nor missed) is constant along diagonals with an overall average of 82% for in-game penalties. A: Percentage saved vs. missed. B: Top10 by percentage of penalties saved.

**Fig 6:**
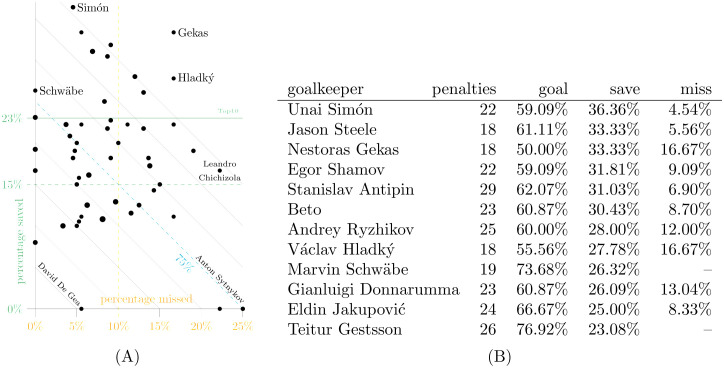
Goalkeepers most frequently facing shoot-out penalties in dataset D3. There are 55 goalkeepers who faced 18 or more shoot-out penalties, and dot size in the scatterplot on the left varies with that number. The overall average goal rate is 75% for shoot-out penalties. A: Percentage saved vs. missed. B: Top10 by percentage of penalties saved.

The situation is qualitatively and quantitatively different for goalkeepers. Because of a routine distinction between penalties saved (for which goalkeepers are praised) and penalties missed (for which kickers are blamed) the analysis below is based on three-way outcome dataset D3. During a match goalkeepers are almost never changed or substituted because of an impending penalty, and very rarely so before a shoot-out. Consequently, they have many more involvements in penalty situations so that we can afford to distinguish between in-game (Fig 5) and shoot-out penalties (Fig 6).

A threshold of 34 yields the 51 goalkeepers facing the highest numbers of in-game penalties. Despite the argument that better goalkeepers make penalty kickers miss more often, we find no correlation between the two rates in general. Goalkeepers are therefore ranked by their save rate. Their collective save rate is 16.02% compared to 13.08% for all others.

While Livakovic has the highest rate of saved in-game penalties, there are three others (Donnarumma, Maignan, and Belenov) who conceded less than 70% because they not only save a high fraction of penalties but kickers also missed at high rates. The majority of frequent goalkeepers has a lower rate of goals against than the average. No one has faced more in-game penalties than Alban Lafont, who saved eight out of 58 penalties.

The same analysis is repeated for goalkeepers facing the highest number of penalties in the shoot-out condition. To obtain a list of 55 goalkeepers, the threshold had to be lowered to 18 shoot-out penalties. Fewer observations are one of the reasons the range of percentages is wider than for in-game penalties.

There is a remarkable line of a dozen goalkeepers who conceded around 60% or fewer penalties in shoot-outs. Their share of saved and missed penalties varies widely from Unai Simón to Leandro Chichizola. Among frequently involved goalkeepers, Anton Sytnykov stands out with an average rate of conceded goals exclusively due to misses, i.e., without saving a single one of them. David De Gea was not quite so lucky, conceding all eleven penalties in the 2021 Europa League final and missing the deciding penalty himself. This 12–11 victory against Manchester United led to the first major trophy for Villareal FC. The single miss against David De Gea occurred in the 2023 FA Cup semifinal against Brighton & Hove Albion FC.

#### Teams taking penalties

Finally, we look at the collective performance of teams during matches, where the choice of kicker is wide open, and in shoot-outs, where selection and preparation feature more prominently.

Only club teams are considered here, because penalties for international sides are even fewer and farther apart.

The thresholds used to obtain at least 50 teams frequently at the mark are 67 for in-game and 25 for shoot-out penalties. This is in line with the aggregate numbers indicating that in-game penalties are twice as frequent as shoot-out penalties.

**Fig 7:**
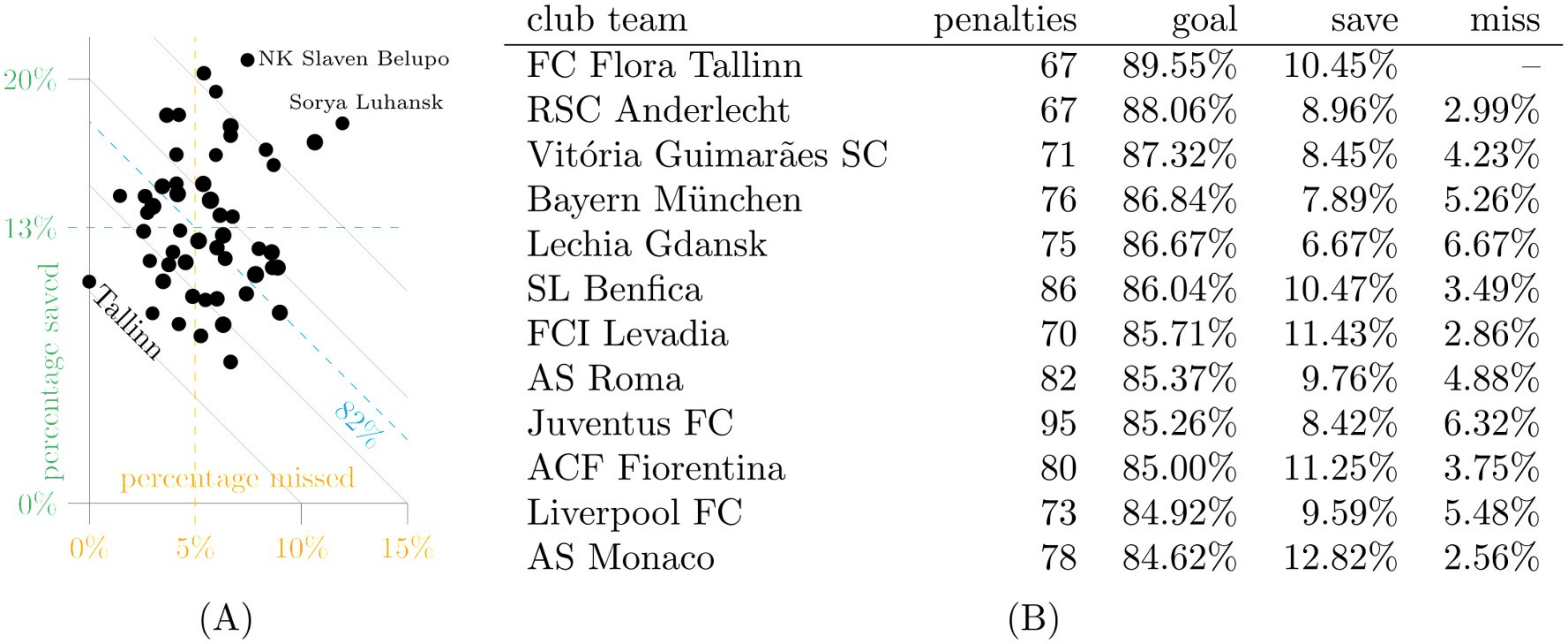
Most frequent teams (minimum of 67 in-game penalties). A: Percentage saved vs. missed. B: Top10 by goal rate.

Unsurprisingly, the data in Fig 7 shows that the most successful teams reduce both save and miss rates, but the large number of collective penalties reduces the advantage over the average penalty taker rather quickly. The combined conversion rate for the 52 most frequently involved teams is 81.16% and thus similar to the rate for all other teams of 82.07%.

**Fig 8:**
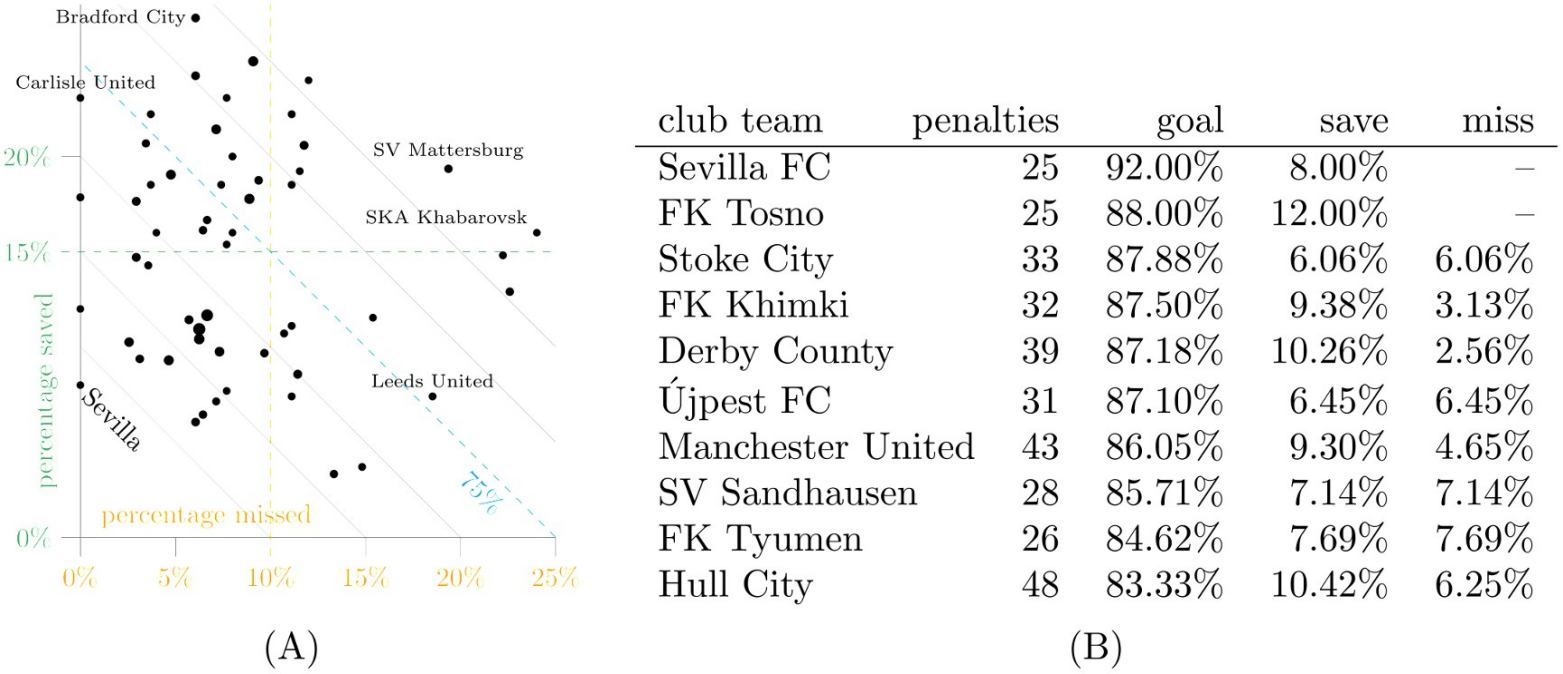
Most frequent teams (25 or more shoot-out penalties). A: Percentage saved vs. missed. B: Top10 by goal rate.

For shoot-out penalties shown in Fig 8, smaller numbers lead to more variation in conversion rates. While teams with many shoot-out penalties do not necessarily score at a higher rate, few of them average more misses than the overall average. Teams such as Carlisle United and Leeds United exhibit average goal rates in very different ways.

Clearly, it helps not to miss any penalty during a shoot-out. When, in addition, a team such as Sevilla FC has only two of 25 penalties saved, chances are they do well in cup competitions. The UEFA Super Cup 2023, in which Sevilla FC lost the penalty shoot-out against Manchester City by missing the last penalty after nine goals, was played after our cut-off date.

The two teams with the highest number of shoot-out penalties in D3 are Chelsea FC and Liverpool FC, taking 64 and 60 penalties, respectively, whereas all other teams took less than 50. These two teams are represented by the largest dots in the lower left quadrant of the scatterplot in Fig 8 and achieved goal rates just below the Top10 despite the high penalty volume.

### Outcomes by moments in time

#### In-game penalties: Match time

**Fig 9:**
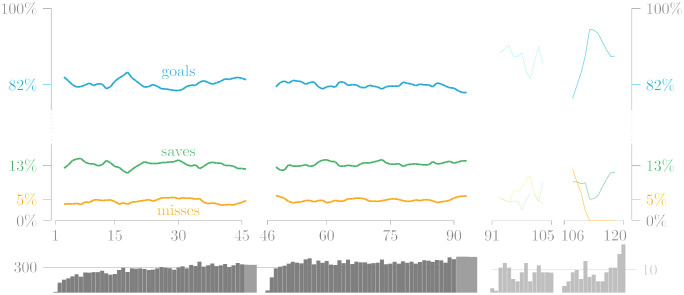
In-game penalty frequencies and three-way outcome rates in dataset D3. For each minute of match time, the 5-minute rolling average of goal, save, and miss rates is shown. Since there is no information on stoppage time in our data, we estimated the true frequency of penalties in the 45th and 90th minute from those of the preceding 44 minutes and spread penalties labeled with the last minute at a constant rate into stoppage. For better comparison, frequency bars in extra time are scaled by the ratio of matches with and without extra time (1:26.88), and rates clearly fluctuate because observations are too few.

In Fig 9, the number of penalties in each minute is plotted against the corresponding conversion rate averaged over surrounding five-minute intervals to smooth the curve and remove timing imprecisions.

Out of 30,908 regulation penalties in D3, 13,047 (42.21%) were awarded in the first half and 17,795 (57.57%) in the second. There are 84 in-game penalties for which time is not available. There is a noticeably low number of penalties in the first two minutes after kick-offs. Since we have no information on the stoppage time in our data, we extrapolated the number of penalties for an estimated stoppage time of three (five) minutes for the first (second) half.

As already apparent from Fig 2, data is very sparse in extra time, and rates fluctuate strongly, re-iterating that large data are needed for reliable results. In regulation, on the other hand, rates are very stable over time and even the difference between the first half (82.39%) and the second half (81.70%) is small.

#### Shoot-out penalties: Alternating order

**Fig 10:**
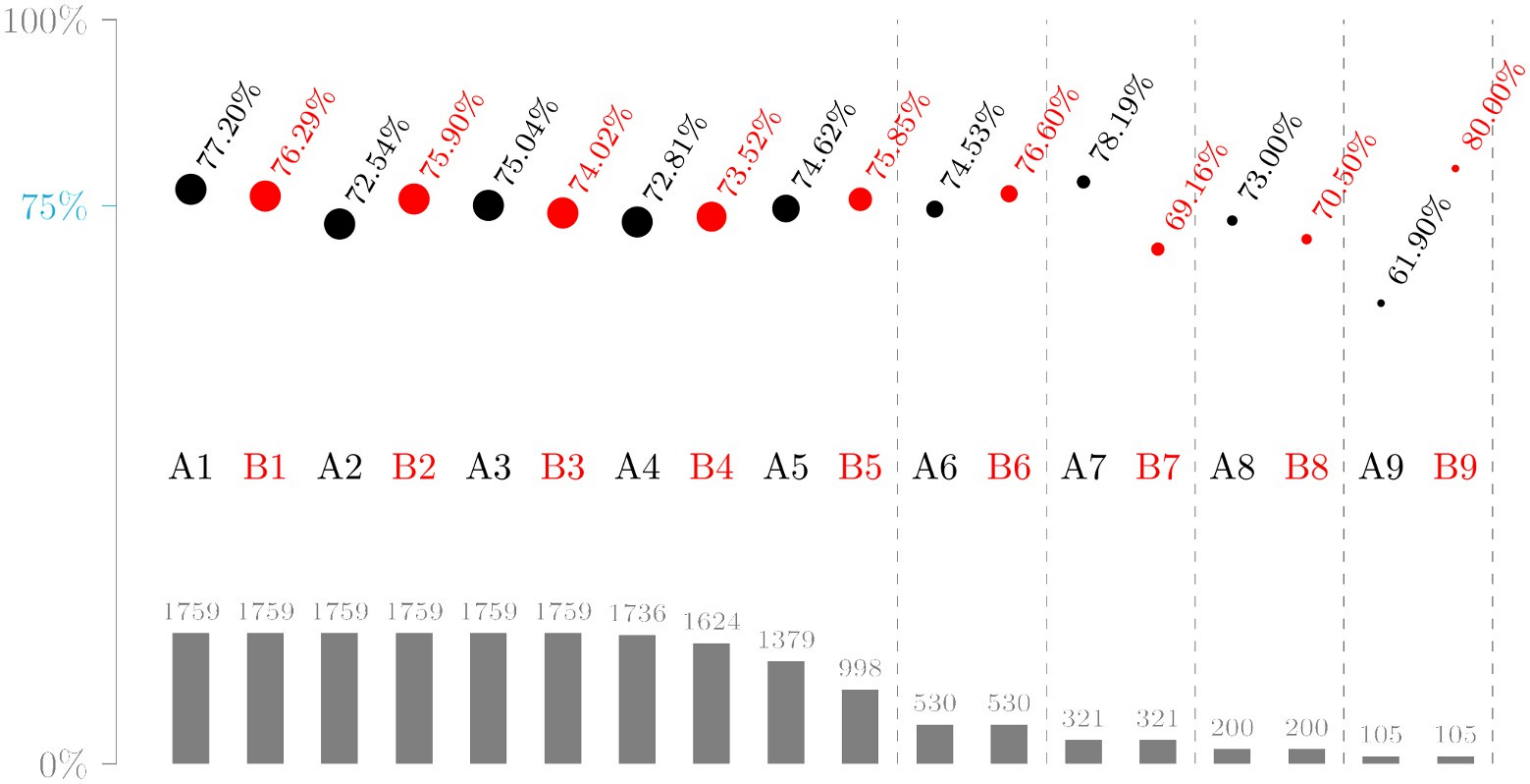
Goal rate by position in 1,759 shoot-outs from dataset D2. Dot areas correspond to the number of penalties given at the bottom.

Success rates for penalty takers split by position in the shooting order are given in Fig 10.

Very few shoot-outs are decided after the minimum of six penalties, the median number is ten, and a third require more than ten. All but 56 shoot-outs in D3 are decided after 18 or fewer penalties. The maximum of 32 penalties was required in three shoot-outs: Scunthorpe United beat Worcester City 14–13 in 2014 (English FA Cup), Derby County beat Carlisle United 14–13 in 2016 (English Football League Cup), and Stade Brestois 29 beat Dinan Léhon FC 13–12 in 2021 (Coupe de France).

On average, the players taking the first two penalties have the highest goal rates. It appears to be a common team strategy to let the most reliable kickers go first [12]. This is corroborated by the observation that the in-game conversion rate of these players is also above average (83.99%). The rate of the second player of the team that starts (A2) is an outlier, and this is largely independent of the score at that moment: 71.47%, 71.77%, or 72.69% if the score is 0–1, 1–0, or 1–1, but 76.77% in 99 cases where it is 0–0. The fifth kicker of the team that follows (B5) does not get to take a penalty in 43.26% of all shoot-outs. Since the number of shoot-outs halves with each subsequent pair of penalties, rates are increasingly fluctuating after the tenth penalty.

**Fig 11:**
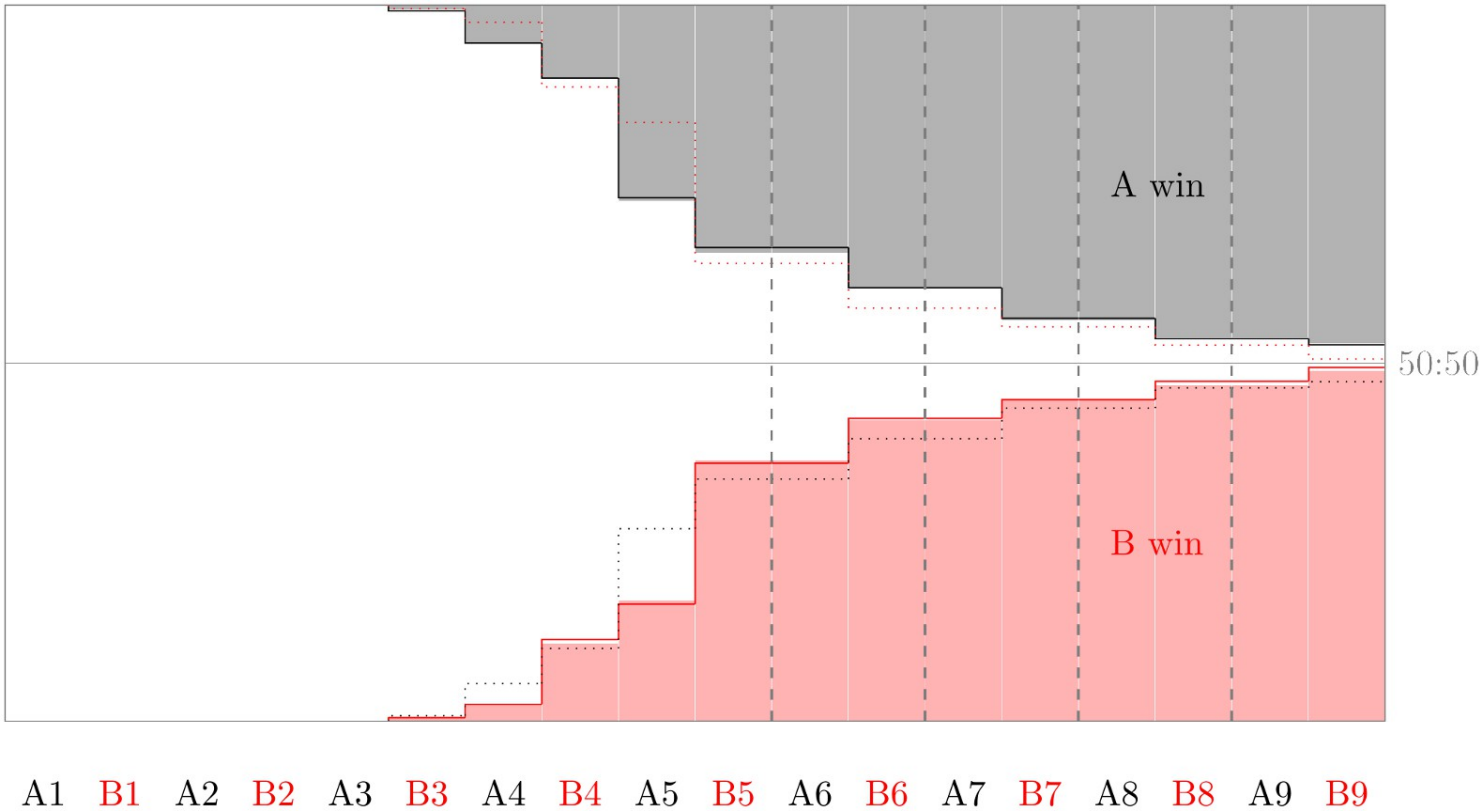
Winning rates within first 18 penalties of 1,759 shoot-outs in dataset D2. Background bars are cumulative winning probabilities derived from independent conversion probabilities according to rates in Fig 10. Solid lines of actual case numbers match them almost perfectly, and are mirrored as dotted lines for better comparison.

The cumulative winning rates of both teams are depicted by solid lines in Fig 11. For comparison, we also calculated the cumulative rates at which teams win, if penalties are converted independently from each other. These are obtained from a Poisson binomial distribution with the observed relative frequencies for each position in the shooting order from Fig 10. The close match of these two curves suggests that scoreline effects, if any, cancel each other out. The starting team (Team A) tends to win earlier, if they win, but as noted above they do not win more often overall. Teams win most often by converting their fifth penalty: Of all shoot-outs in D2, 17% are won by Team A on converting A5 and 20% are won by Team B on converting B5.

In fact, as summarized in Table 7, two thirds of all shoot-outs are decided on a goal (because the other team cannot draw level with the remaining attempts). Note that this is conditional on the fact that this penalty did end the shoot-out, and therefore different from the conversion rate of a potentially last penalty.

**Table 7:** Factually and potentially last penalties compared to others in D2.

situation	penalties	goal
last	1,759	65.55%
not last	17,144	75.57%
potentially winning	1,522	75.76%
potentially losing	2,371	74.44%
neither	15,010	74.56%

In a final comparison, we look at success rates of in-game penalties in relation to the current score (Table 8) and find no significant difference either.

**Table 8:** In-game penalty outcomes in D3 split by current score.

score	penalties	goal	save	miss
in front	8,566	82.38%	12.84%	4.77%
level	13,286	82.07%	13.15%	4.78%
behind	9,330	81.64%	13.64%	4.72%

Information on the current score is missing for 31 penalties.

## Discussion

The results of our analysis suggest three main findings that we discuss below.

### Shoot-outs are tough on the penalty takers

Previous research reports penalty conversion rates between 68% and 85% [2–7, 11–13, 19, 20, 29–33]. While we obtained an overall rate toward the upper end of that interval, it does not seem to be the most relevant number, because we find significant differences between in-game and shoot-out penalties. While in-game penalties are converted 82% of the time, the rate is only 75% for shoot-out penalties. This suggests that the two conditions should be considered separately.

The lower rate in shoot-outs is in line with most previous research [2, 11, 12]. The opposite is found in one study only [13], albeit based on data from only six tournaments. None of the datasets in any of these studies included more than 10,000 penalties, and some of them span multiple decades.

Interestingly, the 7-point performance difference is accounted for by a 5-point increase in misses, and only a 2-point increase in saves. The immediate effect of goalkeeper performance, often considered to be the decisive element in a shoot-out, is thus more limited than it appears. We are not aware of any prior research noting this.

The finding that conversion rates are lower in shoot-outs is robust. It holds even when comparing to in-game penalties restricted to cup competitions, i.e., the settings in which both conditions can occur jointly. If anything, the effect is more pronounced. Outcome rates also do not depend on the level at which leagues are operating, as there are no difference between the Big-5 and other UEFA leagues. It appears that, on average, the quality of penalty takers and goalkeepers vary jointly across leagues. In national cup competitions, participating teams are from different league tiers within a country, so that quality levels of penalty takers and goalkeepers are more varied. The outcome rates shown in Table 3 suggest that this results in an advantage for penalty takers, because in-game goal rates are higher than in leagues. We suspect that this is because better teams with better players have more possession, more box entries, and therefore see more penalties awarded to them, so that quality differences, if any, are in favor of the kicker rather than the goalkeeper. While we did not investigate this further, it will be an interesting direction for future work to study dyadic effects of quality differences more generally.

Assuming that in-game penalties are commonly taken by the most reliable kickers on a team, we should indeed expect that shoot-out penalties have a lower success rate; because of selection constraints, shoot-out penalties are more likely to involve players who would not take a penalty during regulation [8]. We find, however, that this is only part of the explanation, because even those players who are taking in-game penalties see their success rate drop in shoot-outs. Players taking in-game penalties are selected deliberately and often repeatedly, and their in-game goal rate is indeed close to the overall average, as expected. If players taking penalties repeatedly were not significantly more successful on average, their selection would reflect poor decision-making. Their shoot-out success rate, however, is lower by four percentage points, and this is almost entirely due to an increased rate of misses. For players taking only shoot-out penalties, both save and miss rate are higher an additional two percentage points. While the combined four and a half percentage point difference may reflect a quality gap between the two groups of desired and required penalty takers, the performance drop within the first group can be attributed to the shoot-out condition.

Controlling for other factors, the negative and significant effect of the shoot-out condition still stands out in our regression analysis. Other significant effects are related to the type of competition, but confounded with shoot-outs because they feature only in club cup competitions and international-side tournaments and are an indication of competitive balance and high stakes. Interestingly, we find no significant effect for home teams. The higher number of penalties awarded to them is not necessarily unfair, as it may be related to more a attacking playing style at home.

### Fatigue is an unlikely explanation

A commonly offered explanation for a lower number of converted penalties in shoot-outs is that shoot-outs take place at the end of a match—after regulation and, usually, extra time. Many on-field players will have played for 120 minutes by then, and fatigue may reduce their ability to score.

The limited empirical evidence that there has been, however, does not support this reasoning [4, 6]. If fatigue reduces shoot-out performance of penalty takers, we should also observe declining conversion rates over the duration of matches. Where we have sufficient data, however, we find that all three possible outcome rates are very stable over match time. While data is too sparse to conclude that goal rates actually increase in extra time, we certainly find no empirical evidence supporting the idea that fatigue has any noticeable effect on penalty conversion rates.

Similar to previous studies we do find that the rate at which penalties are awarded is increasing over match time [7, 13]. It would therefore be interesting to investigate whether fatigue instead plays a role in this respect.

### There is no first-mover advantage

A second common explanation for lower conversion rates in shoot-outs is that their decisive nature increases the stress level of penalty takers. Stress levels are at the core of debates around the order in which penalties are taken during shoot-outs. Since penalties are more likely to be converted than not, the team following in the alternating order is more often lagging behind. Loss aversion and the prospect of not being able to level would increase stress, and thus lead to an advantage for the team starting.

A number of studies observed this so-called first-mover advantage [19–22], leading to the development of theoretical arguments and the proposal of alternatives [14–18, 34]. Other studies, however, rebuff the existence of a first-mover advantage [8, 23–25].

It should be noted that most of these studies are based on small datasets stretched over very long periods of time. The largest dataset to date covers 1, 623 shoot-outs in national and international competitions from 1970 to 2018 [21]. According to this data, which spans almost five decades, the starting team wins 54.71% of all shoot-outs. In contrast, our dataset D2 contains 1, 759 shoot-outs from the past eleven seasons in competitions all across Europe. According to our data, the starting team wins only 48.83% of shoot-outs. Restricting attention to national cups associated with the top five leagues, the teams going first win 46.98% of 513 shoot-outs. Hence, there is no empirical evidence for a first-mover advantage. Either there is none, or teams have learned to compensate for it.

The intuitive argument of kicker performance being affected by the current score is weakened further as we find virtually no difference in performance between potentially deciding penalties and all others. It appears, therefore, that it is the shoot-out condition as a whole, with its decisive character, heightened attention, and long walk to the spot, that is putting pressure on penalty takers, not fatigue or the current score.

### Limitations

We acknowledge several limitations of our study. In our efforts to account for data inconsistencies and reporting bias, we may have been too eager to remove seeming outliers, and there may be other types of errors that we missed. Both could lead to systematic biases in the outcome rates we report. Since the overall results are consistent with those in the top leagues (where data quality can be expected to be better because of wider attention) we do not expect this problem to be a threat to validity.

We have already emphasized that we do not attempt to analyze techniques to increase penalty success.

Further limitations in scope include our focus on the past eleven seasons. While the data is more recent than in other studies, we cannot make any statement about historical developments or the affect of recent rule changes. In top-level competitions, the now extensive scrutiny of goalkeepers staying on the line might work against them and may have led to increased goal rates. Our initial investigation of the times before and after the introduction of Video Assistant Referees (VAR) in the Big-5 does not point to moderating effects, however.

Except for the FIFA World Cup Finals, we do not cover competitions outside of UEFA. While we found no substantial differences in cursory analyzes of non-European tournaments, more data and checks would be required for proper comparison with other confederations.

Finally, we have consciously excluded women’s football from this study. As we conjecture that qualitatively different insights can be obtained, this should warrant a study in itself.

## Conclusion

We descriptively analysed more than 50,000 penalties from top-division leagues and cups across the Union of European Football Associations (UEFA) over a period of eleven seasons from 2012/2013 to 2022/2023. Approximately one third of these are shoot-out penalties.

Since raw data of more than 60,000 penalties has been obtained from the unofficial records of Transfermarkt, statistical tests based on suspected types of inconsistencies were employed to increase data quality before detailed analyses were performed. For readability and simplicity, we summarize our findings as follows:

- The rate at which penalties are awarded is increasing over the course of a match.
- Four out of five penalties are converted successfully during a match, but only three out of four during shoot-outs. Out of 20 in-game penalties, 16 are converted, three saved and one missed. Out of 20 shoot-out penalties, 15 are converted, three saved and two missed.
- The goal rate of regular penalty takers is four percentage points lower in shoot-outs. This drop in performance is accounted for by additional misses and may therefore be due to pressure on kickers rather than to special skills of goalkeepers.
- The goal rate of non-regular penalty takers appearing only in shoot-outs is lower by another four and a half percentage points. This additional drop is accounted for equally by saves and misses and may therefore be due to player ability.
- In shoot-outs, conversion rates suggest that both teams let their most reliable kickers go first, but also that starting teams select their second kicker differently.
- More than four out of ten shoot-outs are decided before the fifth kicker of the second team gets to take his penalty.
- Two thirds of penalty shoot-outs are decided by a team succeeding rather than failing on the final penalty.
- There is no first-mover advantage in shoot-outs, or it is compensated for. Either way, the alternating shooting order seems fair.

An immediate, if only analytical, consequence of our findings is that in-game and shoot-out penalties should be considered separately in the determination of penalty goal probabilities as used for expected goals in a match. More practical implications mostly concern players taking penalties in shoot-outs:

- In the absence of a scoreline effect, teams can marginally increase their chance of winning by having their best kickers take penalties as early as possible.
- In particular for the starting team it may be beneficial to move the second-most reliable penalty taker to the second position.
- An overwhelming percentage of coaches and players indicated in a survey [19] that they would rather go first. Conveying more clearly to players that there is no advantage may help mitigate the possible effects of over-confidence from the relief of winning the coin toss and stress from losing it.
- The increase in misses among regular penalty takers indicates that players are trying too hard not to have their penalties saved in a shoot-out.
- In light of the performance gap between regular and shoot-out-only penalty takers, teams could consider to rotate in-game execution more widely.

Although the coin toss determining the goal to play on in a penalty shoot-out now seems more important than winning the one to determine the shooting order, there is no choice involved.

It should be borne in mind that we have described large-scale empirical phenomena, and it is at the heart of the attractiveness of the game that anything can happen in a single match or shoot-out, and that there are large variances when considering single players or teams. We hope that our study can serve as a revised baseline to set expectations in modern-day association football. As such it could be helpful for practitioners, commentators, and viewers alike.

## Supporting information

S1 Data (ZIP)
